# Chronic Circadian Rhythm Disturbance Accelerates Knee Cartilage Degeneration in Rats Accompanied by the Activation of the Canonical Wnt/β-Catenin Signaling Pathway

**DOI:** 10.3389/fphar.2021.760988

**Published:** 2021-11-11

**Authors:** Xiaopeng Song, Tianwen Ma, Hailong Hu, Mingchao Zhao, Hui Bai, Xinyu Wang, Lin Liu, Ting Li, Xuanbo Sheng, Xinyu Xu, Xinmin Zhang, Li Gao

**Affiliations:** ^1^ Heilongjiang Key Laboratory for Laboratory Animals and Comparative Medicine, College of Veterinary Medicine, Northeast Agriculture University, Harbin, China; ^2^ College of Veterinary Medicine, Northeast Agricultural University, Harbin, China

**Keywords:** circadian rhythm disturbance, osteoarthritis, wnt/β-catenin signaling pathway, cartilage, rat

## Abstract

With the gradual deepening of understanding of systemic health and quality of life, the factors affecting osteoarthritis (OA) are not limited to mechanical injury, metabolic abnormality, age and obesity, etc., but circadian rhythm, which plays a non-negligible role in human daily life. The purpose of this study was to explore the molecular mechanism of chronic circadian rhythm disturbance (CRD) inducing cartilage OA-like degeneration. Rats with the anterior cruciate ligament excision transection (ACLT) were used to establish the early-stage OA model (6-week). The light/dark (LD) cycle shifted 12 h per week for 22 weeks in order to establish a chronic CRD model. *BMAL1* knockdown (KD) and Wnt/β-catenin pathway inhibition were performed in chondrocytes. The contents of proinflammatory factors and OA biomarkers in serum and chondrocyte secretions were detected by ELISA. Pathological and immunohistochemical staining of articular cartilage indicated the deterioration of cartilage. WB and qPCR were used to evaluate the relationship between matrix degradation and the activation of Wnt/β-catenin signaling pathway in chondrocytes. We found that chronic CRD could cause OA-like pathological changes in knee cartilage of rats, accelerating cartilage matrix degradation and synovial inflammation. The expression of MMP-3, MMP-13, ADAMTS-4, and β-catenin increased significantly; BMAL1, Aggrecan, and COL2A1 decreased significantly in either LD-shifted cartilage or *BMAL1*-KD chondrocytes. The expression of β-catenin and p-GSK-3β elevated, while p-β-catenin and GSK-3β diminished. The inhibitor XAV-939 was able to mitigated the increased inflammation produced by transfected *siBMAL1*. Our study demonstrates that chronic CRD disrupts the balance of matrix synthesis and catabolic metabolism in cartilage and chondrocytes, and it is related to the activation of the canonical Wnt/β-catenin signaling pathway.

## Introduction

Environmental factors can significantly affect body metabolism, especially light, temperature and humidity. Light/dark (LD) cycle is the most important environmental zeitgeber that affects circadian homeostasis. Irregular LD cycles can disrupt the body’s circadian rhythm, even metabolic disorders, especially in shift workers (Wright et al., 2013). Previous studies have suggested that shift work increased the risk of chronic metabolic-related diseases, such as cardiovascular disease and type 2 diabetes (Hublin et al., 2010; Shan et al., 2018), and one common risk factor shared by these pathologies is inflammation (Castanon-Cervantes et al., 2010). Chronic circadian rhythm disturbance (CRD) could significantly affect the inflammatory response, manifested as the abnormal diurnal variation of proinflammatory factors, such as TNF-α and IL-6 (Vgontzas et al., 2004). Zhou et al. has proposed that shift work was an independent risk factor for knee osteoarthritis (OA) (Zhou et al., 2020). A recent study found that 24 h continuous light induced an inflammatory microenvironment in osteoarthritic joints, and even developed osteoporosis (Hong et al., 2019). Moreover, Kc et al. found OA-like pathological changes in the mouse knee joint by conducting long-term (22 weeks) disruption of the circadian environment (Kc et al., 2015; Kc et al., 2015). These suggest that the occurrence and development of OA are closely related to the biological clock system.

Clock genes are involved in the metabolic pathways of bone and cartilage, and many studies have confirmed the rhythmic changes in cartilage metabolism (Kaoru et al., 2013; Andersson et al., 2006; Kong et al., 2006). As a core clock protein, the expression of brain and muscle ARNT-like 1/Arntl (BMAL1) in OA joints is low, and is inversely proportional to the severity and progression of OA (Dudek et al., 2016; Akagi et al., 2017). Samsa et al. (2016) demonstrated progressive joint injury and systemic low bone mass in *BMAL1* ^−/−^ mouse joints. Additionally, BMAL1 can regulate the activity of chondrocytes through HIF1α-VEGF and TGF-β signaling pathways (Akagi et al., 2017; Ma et al., 2019). The canonical Wnt/β-catenin signaling pathway, as one of the most important pathways related to cell proliferation and development, is also critical to the occurrence and development of OA. The classical Wnt/β-catenin pathway was activated in the chondrocytes of OA patients, resulting in increased expression of matrix metalloproteinases (MMPs) and A disintegrin and metalloproteinase with thrombospondin motifs (ADAMTS), inducing degradation of extracellular matrix (ECM), and exacerbating cartilage damage (Ning et al., 2012). OA-like changes were also found in transgenic mice with high β-catenin specific expression, which directly demonstrated the role of Wnt/β-catenin pathway in OA (Zhu et al., 2009).

Numerous studies have shown that clock genes are involved in activation and inhibition of the Wnt/β-catenin pathway (Guo et al., 2012; He et al., 2013; Lin et al., 2013; Li et al., 2017; Li et al., 2021). Knockdown Period 3 (PER3) accumulated BMAL1 and p-β-catenin protein, and promoted tumor formation. After overexpressing *BMAL1* in NIH-3T3 cells, Lin et al. found a significant increase in the expression of β-catenin, suggesting that the activation of the Wnt pathway may be the mechanism by which the clock gene *BMAL1* promotes cell proliferation. Additionally, *BMAL1* gene plays a synergistic role with the Wnt/β-catenin signaling pathway in adipogenic and osteogenic differentiation of mesenchymal stem cells (MSCs). However, these illustrate an interesting phenomenon that these theories are contrary to the previously proposed expression of BMAL1 and the Wnt pathway in OA cartilage. Therefore, can BMAL1 still cooperates with Wnt/β-catenin pathway in OA? Or whether it’s relevant? In this study, we established chronic CRD models in rats and chondrocytes, in order to study the effects of CRD on rat articular cartilage and the relation between clock proteins and the Wnt/β-catenin pathway.

## Materials and Methods

### Antibodies

Antibodies used for western blotting (WB) and immunostaining included mouse anti- COL2A1 (Novus; United States), rabbit anti-β-catenin (ABclonal; China), rabbit anti-BMAL1 (Absin; China), rabbit anti-MMP-3 (CST; United States), rabbit anti-MMP-13 (ABclonal; China), rabbit anti-ADAMTS-4 (ABclonal; United States), rabbit anti-Cyclooxygenase-2 (COX-2) (CST; United States), rabbit anti-β-catenin (ABclonal; China), rabbit anti-GSK-3β (ABclonal; China), mouse anti-p-β-catenin (SANTA; United States), mouse anti-p-GSK-3β (SANTA; United States), mouse anti-GAPDH (Zsbio; China), Alexa Fluor® 488-conjugated goat anti-mouse immunoglobulin G (IgG) (H + L) (Servicebio; China), Cy3 conjugated goat anti-rabbit IgG (H + L) (Servicebio; China), Horseradish Peroxidase (HRP) goat anti-mouse IgG (H + L) (Zsbio; China), and HRP goat anti-rabbit IgG (H + L) (Zsbio; China).

### Cell Culture and Small Interfering RNAs Transfection

Primary chondrocytes were isolated from tibial platform of rats under sterile conditions, released by treating with 0.25% Trypsin-EDTA and 0.2% Type II collagenase (Gibco Company, United States) at 37°C (5% CO2) in Dulbecco’s Modified Eagle’s Medium (DMEM)/Hams F-12, supplemented with 10% fetal bovine serum (FBS) (BI, Israel) and 1% Penicillin/Streptomycin. After passage, a second generation of chondrocytes was used for the experiment. For cell transfection, chondrocytes in logarithmic growth phase were taken and transfected when the density reached 60–80%. GP-Transfect-Mate medium mixture (serum-free medium + GP-Transfect-Mate) and *siRNA* medium mixture (serum-free medium + siBMAL1 (20 μM)) (Genepharma, China) were prepared and transfected. Sequences for negative control and *siBMAL1s* are listed in Table 1. DMEM/F-12 containing 10% FBS was replaced after 4 h, and the result was tested by WB or qPCR at 72 h after transfection.

**TABLE 1:** Three siBMAL1 sequences and negative control sequence for the experiment.

Name	Sequences (5′→3′)
Negative control	Sense:UUCUCCGAACGUGUCACGUTT
	Antisense:ACGUGACACGUUCGUAGAATT
BMAL1siRNA-614	Sense:CCUCCACAAUCAGUGACUUTT
	Antisense:AAGUCACUGAUUGUGGAGGTT
BMAL1siRNA-1083	Sense:CCGAGGGAAGAUCCUCUUUTT
	Antisense:AAAGAGGAUCUUCCCUCGGTT
BMAL1siRNA-1125	Sense:CCUCAAUUAUAGCCAGAAUTT
	Antisense:AUUCUGGCUAUAAUUGAGGTT

### Immunofluorescence Assay

The secondary generation chondrocytes were diluted to 2×10^4^ cells/ml, and cultured in 6-well plates. Cells were fixed with 4% paraformaldehyde, permeabilized, and then blocked with 3% bovine serum albumin (BSA) at room temperature for 30 min. After removing the blocking solution, chondrocytes were incubated with primary antibody overnight at 4°C. COL2A1 (1:100) and DAPI for chondrocyte identification, β-catenin (1:100) for nuclear translocation. The cell climbing slides were washed and covered with fluorescently labeled secondary antibody for 50 min at room temperature. DAPI was incubated for 10 min at dark. Fluorescence was observed under a fluorescence microscope (Nikon Eclipse Ti-S; Japan) at 545 nm (red) and 340 nm (blue).

### Real-Time PCR

Ribonucleic acid (RNA) in the chondrocytes was extracted at 4°C using SV total RNA isolation system (Promega, United States), the concentration was measured at 260 and 280 nm, and then reverse transcribed blending with GoScript™ Reverse Transcription Mix, Oligo (dT) (Promega, United States) into cDNA (1 μg/ml). The mixture of 10 μl GoTaq®qPCR Master Mix, 0.6 μl forward and reverse primers, 2.5 μl cDNA and 6.3 μl RNase-free ddH_2_O (Promega, United States) were subjected to 40 amplification cycles. Primer sequences are listed in Table 2. The intensity of fluorescence signal accumulation during the entire PCR process was monitored using LightCycler^®^480 (Roche, Germany) to obtain the cycle threshold (Ct).

**TABLE 2:** Primer sequences for the experiment.

Name	Sequences (5′→3′)
*GAPDH*	F:GATGCCCCCATGTTTGTGAT
	R:GGCATGGACTGTGGTCATGAG
*MMP-3*	F:TTCTGGTCTTCTGGCACACG
	R:GCATCGATCTTCTGGACGGT
*MMP-13*	F:TTCTGGTCTTCTGGCACACG
	R:TGGAGCTGCTTGTCCAGGT
*ADAMTS-4*	F:CACCGAACCGACCTCTTCAA
	R:GAGTTCCATCTGCCACCCGT
*BMAL1*	F:CAGAAGCAAACTACAAGCCAA
	R:GGTCACATCCTACGACAAACA
*GSK-3β*	F:AGCTGTATTAAAACTCTGCGACT
	R:TTGGGTTCATTTCTCTAATTTGCT
*β-catenin*	F:CAGTTCGCCTTCACTATGGA
	RGGGCAAGATTTCGAATCAAT
*COX-2*	F:AGAAGCGAGGACCTGGGTTCAG
	R:ACACCTCTCCACCGATGACCTG
*COL2A1*	F:GAACGGCGGCTTCCACTTCAG
	R:GCTTCGTCCAGGTAGGCAATGC
*Aggrecan*	F:CACACGCTAGACACTGGACT
	R:TCACACTGGTGGAAGCCATC

### Sodium Dodecyl Sulfate -Polyacrylamide Gel Electrophoresis and WB

Total protein samples of chondrocytes were extracted following the instructions (Beyotime; China). The mixture of radio immunoprecipitation assay (RIPA) and Phenylmethylsulfonyl fluoride (PMSF) fully lysed the cells. Then equal 20 μg amounts of protein were separated onto 10% SDS-PAGE and transferred to nitrocellulose (NC) filter membrane. The membranes were blocked in 5% nonfat milk dissolved in Tris Buffered Saline with Tween®20 (TBST) buffer and probed with primary antibodies for BMAL1 (1:1,500), MMP-3 (1:1,000), MMP-13 (1:1,000), ADAMTS-4 (1:1,500), COX-2 (1:1,000), β-catenin (1:2,000), GSK-3β (1:1,500), p-β-catenin (1:1,000), p-GSK-3β (1:1,000), and GAPDH (1:3,000) at 4°C overnight followed by secondary antibody coupled with horseradish peroxidase at room temperature for 2 h. The blots were developed using the ECL system (Tanon-5200; China) and the gray value was quantified with Image J software (1.53c; United States).

### *In vivo* Animals and OA Induction

Adolescent (8–10 week old) Sprague Dawley rats were purchased from Liaoning Changsheng biotechnology co.,Ltd. The applicable Johns Hopkins Bloomberg School of Public Health Guidelines for the care and use of animals were followed. The ARRIVE (Animal Research: Reporting *In Vivo* Experiments) guidelines to improve standards of reporting of animal experiments was used. The experiment was divided into four groups: Control group; OA group; Shift group; and Non-shift group.

For OA model, the anterior cruciate ligament excision transection (ACLT) to the experimental rats was used according to the modeling method summarized by Teeple et al. (2013). Rats were narcotized with isoflurane and immobilized in the supine position. An incision of 1–1.5 cm in length was cut in the longitudinal direction of the surgical department to expose articular cavity and the ACL was broken by micro-shearing without damaging the articular cartilage under a microsurgical microscope. After performing a drawer experiment to confirm the surgery, the joint capsule and skin were sutured. The control (sham) group only cut the skin and subcutaneous fascia in the surgical department, did not expose the joint cavity, and then sutured. Mice were sacrificed at the end of the sixth week. The entire knee joints were harvested and submitted for histological analyses. The serum was collected for subsequent ELISA tests.

### Chronic Environmental Disruption of Circadian Rhythm Protocol

**FIGURE 1:**
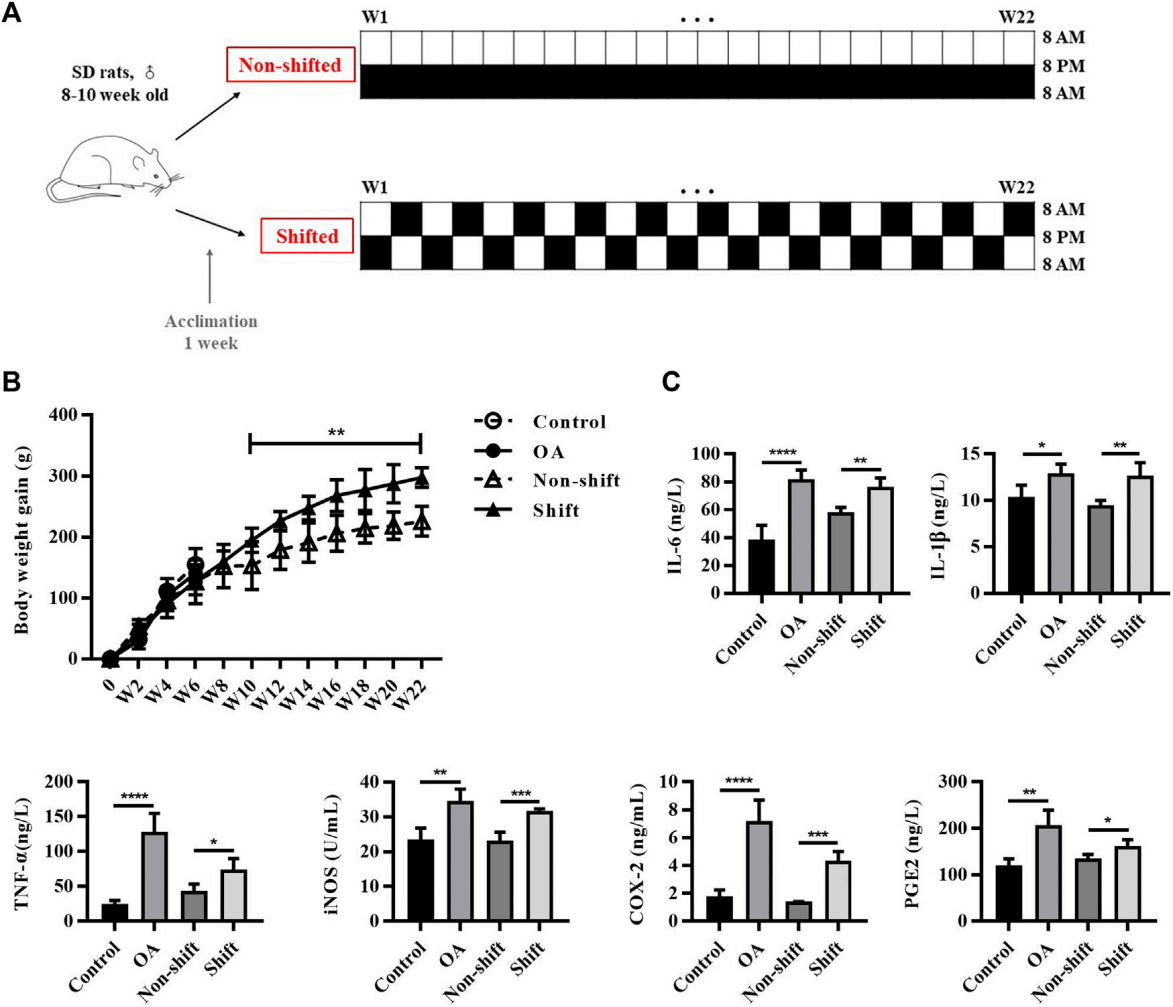
Protocol of CRD-rat model and inflammatory response in rats after OA and CRD modeling. **(A)** Protocol of CRD-rat model and the control group. **(B)** Alterations of body weight gain of rats in different groups. Results are expressed as mean ± SD; *n* = 4–6. ^**^ *p* < 0.01 between the shift and non-shift group. **(C)** Levels of IL-1β, TNF-α, IL-6, iNOS, COX-2, and PGE2 in rat serum in different groups. Results are expressed as mean ± SD; *n* = 4–6. ^*^ *p* < 0.05, ^**^ *p* < 0.01, ^***^ *p* < 0.001 and ^****^ *p* < 0.0001.

The OA model was not performed in the chronic shift group and the non-shift group. The modeling method of chronic CRD summarized by Preuss et al. (Preuss et al., 2008) was adopted. The non-shift (control) group maintained a constant L/D (12/12) (L: 8 AM-8 PM; D: 8 PM-8 AM) cycle during the entire experiment. The chronic shift model group also maintained a constant L/D (12/12) cycle, but with a 12 h phase shift per week for 22 weeks **(** Figure 1A). The rats were constantly entering new LD cycles, resulting in chronic CRD. The rat knee joints were harvested at the end of the 22nd week after sacrificed. Subsequent studies were consistent with those of OA rats.

### Histological Staining and Immunohistochemical Analysis

Bones were dehydrated and waxed in a gradient alcohol. After the wax-soaked tissues were blocked, sliced the trimmed wax block in a paraffin section, 4 μm. The water is roasted with dry wax and stored at room temperature. Paraffin slides dewaxed as follows: Two changes of pure xylene-two changes of pure ethanol-75% ethanol. For special staining, the slides stained in fast green dye solution for 1–5 min, washed away the excess dye solution until the cartilage was colorless, and soaked in 1% hydrochloric acid and alcohol for 10 s. Then staining with saffron dye solution for 1–5 s, and rapid dehydration in absolute ethanol. The slides were immersed in xylene to transparent, sealing with neutral resin. Red or orange-red cartilage and green bone formation were observed under microscope (Nikon E100; Japan), and took images for analysis. The scoring criteria for OA cartilage pathology were based on the assessment system issued by Osteoarthritis Research Society International (OARSI) (Pritzker et al., 2006).

For immunohistochemical analysis, deparaffinzing and rehydrating paraffin sections were performed following by antigen retrieval, blocking endogenous peroxidase activity and serum sealing. Incubations were performed overnight at 4°C with primary antibodies for BMAL1 (1:100), MMP-3 (1:100), MMP-13 (1:100), ADAMTS-4 (1:200), β-catenin (1:100), Aggrecan (1:100), and COL2A1 (1:20) prepared with PBS. Then the tissues were covered with secondary antibody (HRP labeled) and incubated at room temperature for 1.5 h. After Diaminobenzidine (DAB) chromogenic reaction and nucleus counterstaining with hematoxylin stain solution, the slices were dehydrated and sealed following by examined under a microscope (Nikon E100; Japan) and analyzed.

### Enzyme-Linked Immunosorbent Assay of Serum and Extracellular Secretions

The serum and extracellular secretions (ECS) was extracted after centrifugation (2,500 rpm/min, 20 min). ELISA was performed following the instructions strictly by using rat tumor necrosis factor α (TNF-α) (MEIMIAN, China), Interleukin-6 (IL-6) (MEIMIAN, China), C-terminal crosslinking telopeptide of type Ⅱ collagen (CTX-Ⅱ) (MEIMIAN, China), cartilage oligomeric matrix protein (COMP) (MEIMIAN, China), inducible Nitric Oxide synthase (iNOS), prostaglandin E2 (PGE2), and cyclooxygenase-2 (COX-2) (NJJCBIO, China) ELISA kit. The absorbance of each tube was measured with a microplate reader at 450 nm reflecting the content and activity. ELISAcalc software was used to calculate the regression equation of the standard curve linear according to the concentration and OD value and four-parameter logistic curve was selected for fitting model.

### Statistical Analysis

Data are presented as mean ± SD *in vivo* and mean ± S.E.M *in vitro*. Unpaired t test was performed between the two groups. When three or more groups are compared, IBM SPSS Statistics 25.0 with one-way ANOVA and Tukey test for post hoc test were used. Graphs were drawn using the GraphPad Prism (version 8). Values with *p* < 0.05 were considered statistically significant, and *p* < 0.01 extremely significant.

## Results

### Chronic CRD can Induce Inflammation Similar to OA in Rats

Epidemiological investigation showed that long-term shift workers were more prone to obesity (Kubo et al., 2011), and obesity is also one of the major causes of OA. In this study, we monitored the body weight of rats, and found abnormal increase in shift group. No significant difference in body weight gain was found between control and OA group in 6 weeks. However, from week 10 to week 22, the weight gain of shift group was significantly higher than that of non-shift group (*p* < 0.01) (Figure 1B). By evaluating the changes of pro-inflammatory cytokines in circulating blood, the levels of IL-1β, IL-6, TNF-α, iNOS, COX-2 and PGE2 of OA rats were significantly increased compared with the control group (*p* < 0.05, *p* < 0.01, and *p* < 0.0001). Similarly, compared with non-shift rats, the shift group showed a similar upward trend of these inflammatory markers (*p* < 0.05, *p* < 0.01, and *p* < 0.001) (Figure 1C). It suggests that CRD can induce inflammation in rats.

### Chronic CRD Alters Matrix Degradation of Cartilage and Clock Protein Expression

**FIGURE 2:**
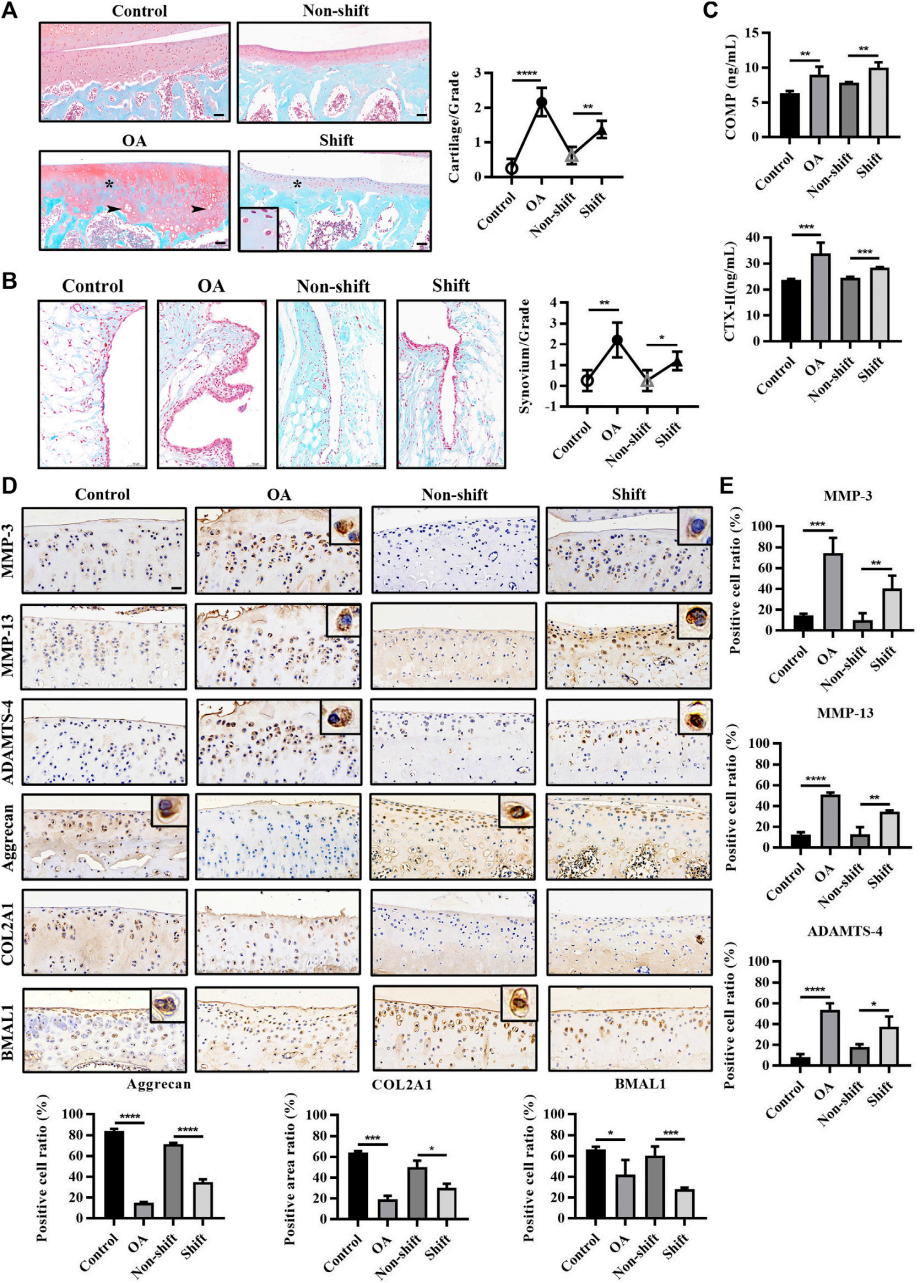
Pathological changes and immunological detection of knee cartilage in each group. **(A)** Safranine O and fast green staining of rat tibia cartilage and OARSI cartilage score. Arrows, vacuolar and hypertrophic chondrocytes; asterisk, absence of cationic staining. Magnification (Mag), 200×. Scale bar, 50 μm. Results are expressed as mean ± SD; *n* = 4–6. **(B)** Safranine O and fast green staining of rat knee synovium. Mag, 200×. Scale bar, 50 μm. Results are expressed as mean ± SD; *n* = 3–6. **(C)** Levels of COMP and CTX-Ⅱ in rat serum in each group. Results are expressed as mean ± SD; *n* = 4–6. **(D,E)** Immunohistochemical staining of MMP-3, MMP-13, ADAMTS-4, Aggrecan, COL2A1, and BMAL1 in chondrocytes. Mag, 200×. Scale bar, 50 μm. Results are expressed as mean ± SD; *n* = 4. *p < 0.05, **p < 0.01, ***p < 0.001, and ****p < 0.0001.

To assess the effect of CRD on the articular cartilage, histological staining and immunological assays of the tibial plateau were performed. As shown in Figure 2A, the surface of cartilage in the control and non-shift group was smooth, and the matrix was evenly red stained. In the OA and Shift groups, the surface layer was rough, and red staining was decreased (asterisk), indicating the reduction of cartilage matrix synthesis or increase of degradation. However, hypertrophic and vacuolar chondrocytes could be seen (arrows) in OA cartilage but not in shift group. Additionally, OA and shift rats showed significant hyperplasia of synovium lining cell layer compared with control and non-shift rats, and inflammatory cells accumulated in small quantities (Figure 2B). Meanwhile, the OA biomarkers COMP and CTX-Ⅱ were significantly increased in OA rats and shift rats (Figure 2C). These indicate that the OA-like pathological changes of cartilage can be induced by altering the LD cycle for a long time.

Additionally, the significant increase of some hydrolytic proteases (MMP-3, MMP-13 and ADAMTS-4) (*p* < 0.001 and *p* < 0.0001) and the significant decrease of matrix fibers (Aggrecan and COL2A1) (*p* < 0.001 and *p* < 0.0001) in OA cartilage indicated that the inflammatory response of OA cartilage was severe. Interestingly, similar changes in protein expression were observed in the shift group (Figures 2D,E) (*p* < 0.05, *p* < 0.01, *p* < 0.001, and *p* < 0.0001), which suggested that LD cycle disturbance bias the rate of cartilage degradation over matrix synthesis. Both ACLT and CRD significantly reduced the expression of BMAL1 in cartilage (Figures 2D,E) (*p* < 0.05 and *p* < 0.001), indicating the dysregulation of cartilage clock.

### Knockdown-BMAL1 Stimulates an Inflammatory Response in Chondrocytes

**FIGURE 3:**
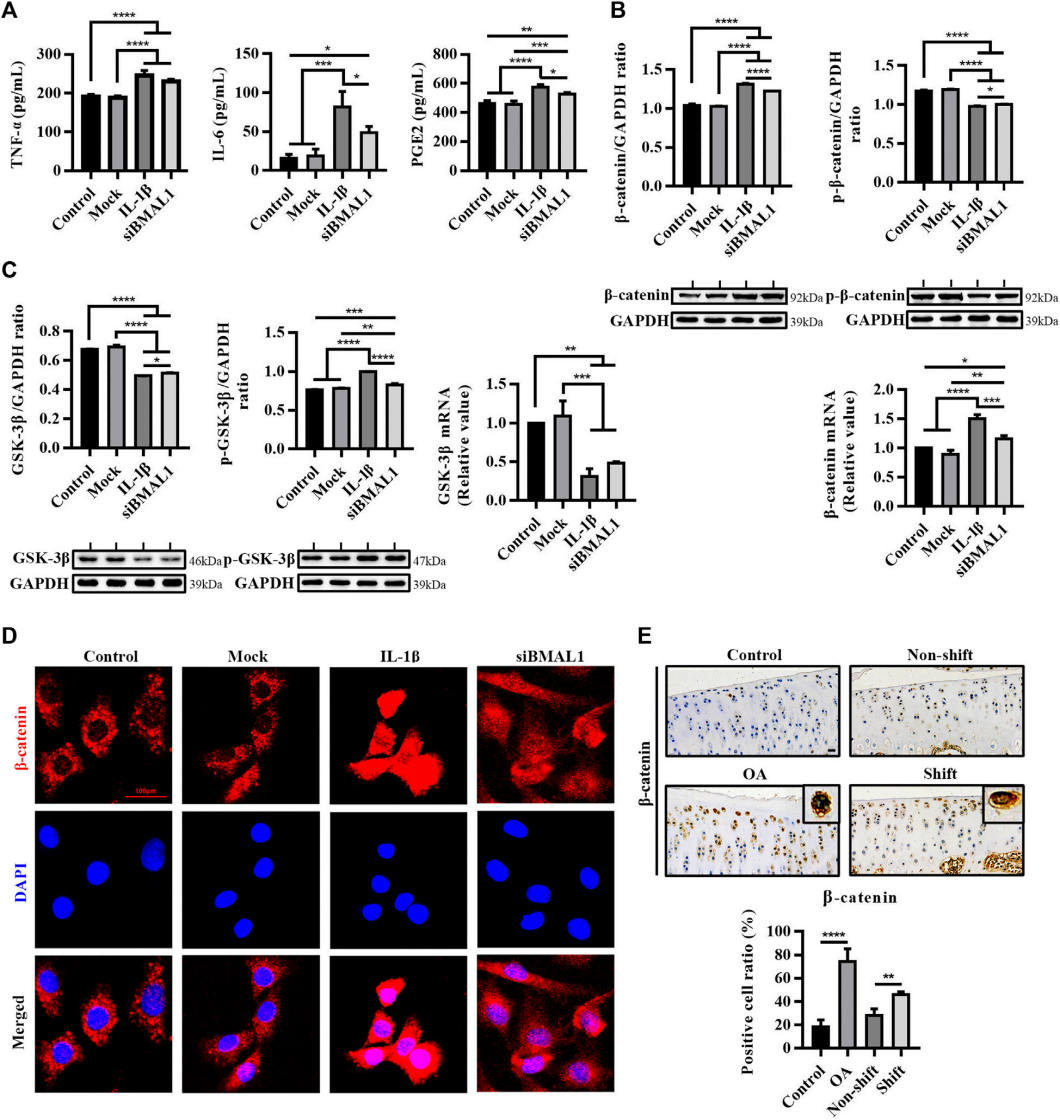
Inflammatory response and activation of the WNT/β-catenin pathway *in vitro* chondrocytes stimulated by IL-1β and siBMAL1. **(A)** Levels of TNF-α, IL-6, and PGE2 in ECS. Results are expressed as mean ± SEM; *n* = 3–4. **(B)** Protein expression changes of β-catenin and p-β-catenin, and mRNA levels of β-catenin in chondrocytes in each group. All experiments were performed in triplicate, and the results are expressed as the mean ± SEM. **(C)** Protein expression changes of GSK-3β and p-GSK-3β, and mRNA levels of GSK-3β in chondrocytes in each group. All experiments were performed in triplicate, and the results are expressed as the mean ± SEM. **(D)** Nuclear translocation of β-catenin in chondrocytes stimulated with IL-1β and transfected with siBMAL1. β-catenin was highly expressed in the cytoplasm of control chondrocytes, yet mostly transferred into the nucleus after IL-1β stimulation. A considerable number of β-catenin protein were also transferred to the nucleus in KD-BMAL1 chondrocytes. Mag, 400×. Scale bar, 100 μm. **(E)** Expression changes of β-catenin in tibial plateau cartilage of rats in each group. Mag, 200×. Scale bar, 50 μm. Results are expressed as mean ± SD; *n* = 4. *p < 0.05, **p < 0.01, ***p < 0.001, and ****p < 0.0001.

In order to further explore the underlying mechanism of cartilage clock disorder, we knocked down the BMAL1 protein in second-generation chondrocytes (Supplementary Figure S1). All three siBMAL1s significantly reduced the expression of BMAL1 protein in chondrocytes, with interference efficiency between 30 and 40% (Supplementary Figure S2A). And compared with the control group, no significant change of BMAL1 expression in negative control (mock) group was found. In order to improve the efficiency of siRNA interference, a hybrid siBMAL1 interference (siRNA614:siRNA1083:siRNA1125 = 1:1:1) was performed. Protein silencing efficiency was about 50% (*p* < 0.0001) (Supplementary Figure S2B), and gene silencing efficiency was about 80% (*p* < 0.0001) (Supplementary Figure S2C). *In vitro* OA chondrocyte model was established by treating chondrocytes with 10 ng/ml IL-1β (Wu et al., 2018). The secretion of IL-6, TNF-α, and PGE2 was significantly increased after treatment with IL-1β and siBMAL1 compared with the control group (Figure 3A). It is suggested that inhibition of BMAL1 expression can produce an inflammatory response similar to IL-1β stimulation.

### Inhibition of BMAL1 Activates the Wnt/β-Catenin Pathway in Chondrocytes

Two studies have shown that IL-1β activates the Wnt/β-catenin signaling pathway in chondrocytes (Wu et al., 2018; Xi et al., 2020). In this study, the expression of β-catenin protein and mRNA in chondrocytes treated with IL-1β were significantly increased (*p* < 0.0001), and the expression of p-β-catenin was significantly decreased (*p* < 0.0001), compared with the control group. Similarly, a similar trend occurred after silencing BMAL1 (*p* < 0.0001) (Figure 3B). The protein and mRNA contents of GSK-3β were significantly dampened after IL-1β stimulation (*p* < 0.0001), BMAL1-KD also accelerated the phosphorylation of GSK-3β (*p* < 0.01) (Figure 3C). Moreover, IL-1β and siBMAL1 both induced different degrees of nuclear translocation of β-catenin in chondrocytes (Figure 3D). These indicate that the down-regulation of BMAL1 expression activates the Wnt/β-catenin signaling pathway.

*In vivo*, the percentage of β-catenin positive cells in the OA and Shift groups was extremely significantly ascended compared to the control group (Figure 3E). It is suggested that the disturbance of the cartilage clock may induce the expression of a series of downstream inflammatory factors and matrix degradation enzymes by activating the Wnt/β-catenin signaling pathway, thus accelerating the degradation of cartilage.

### Suppression of Wnt/β-Catenin Pathway Alleviates the Degradation of Chondrocyte Matrix Caused by siBMAL1

**FIGURE 4:**
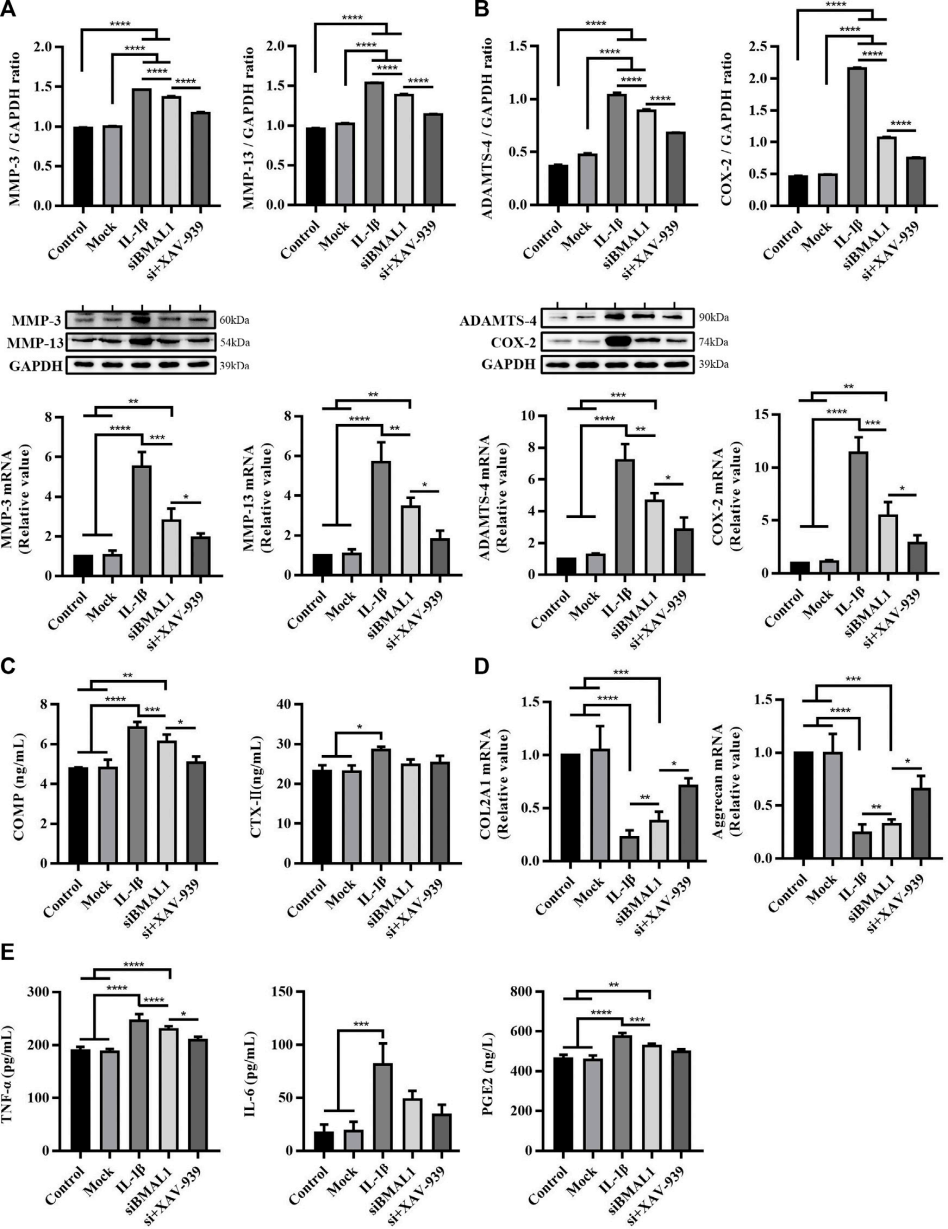
Cartilage matrix degradation and inflammation response in chondrocytes after stimulating with IL-1β, transfecting with siBMAL1 and suppressing β-catenin pathway. **(A)** Protein expression and mRNA level changes of MMP-3 and MMP-13 in chondrocytes in each group. **(B)** Protein expression and mRNA levels changes of ADAMTS-4 and COX-2 in chondrocytes in each group. **(C)** Levels of COMP and CTX-Ⅱ in ECS. **(D)** mRNA level changes of COL2A1 and Aggrecan in chondrocytes in different groups. **(E)** Levels of TNF-α, IL-6, and PGE2 in ECS. All experiments were performed in triplicate, and the results are expressed as the mean ± SEM. *p < 0.05, **p < 0.01, ***p < 0.001, and ****p < 0.0001.

As can be seen from Figures 4A,B, compared with the control group and mock group, the expression levels of MMP-3, MMP-13, ADAMTS-4, and COX-2 protein and corresponding mRNA levels in chondrocytes treated with IL-1β and siBMAL1 were significantly increased (*p* < 0.01, *p* < 0.001, and *p* < 0.0001). This corresponds to the results *in vivo*. The Wnt/β-catenin pathway specific inhibitor XAV-939 has been shown to be significantly effective in improving an OA-mouse model (Lietman et al., 2018). After co-treatment with XAV-939, the expression levels of these proteins and mRNAs were significantly decreased compared with siBMAL1 group (*p* < 0.05 and *p* < 0.0001) (Figures 4A,B). XAV-939 also significantly dampened the increase of COMP caused by siBMAL1 (*p* < 0.05), but had no obvious effect on CTX-Ⅱ (Figure 4C). These findings suggest that the canonical Wnt/β-catenin pathway plays an important role in the mechanism of accelerated matrix degradation caused by disruption of cartilage clock.

Additionally, the contents of COL2A1 and Aggrecan mRNA in cells were extremely decreased after stimulation of IL-1β and transfection of siBMAL1 (*p* < 0.001 and *p* < 0.0001). XAV-939 significantly recovered the reduced COL2A1 and Aggrecan mRNA (*p* < 0.05) (Figure 4D), promoting chondrocyte matrix synthesis. In addition, levels of pro-inflammatory factors in ECS following inhibition of the Wnt/β-catenin pathway were also detected. The results showed that XAV-939 significantly reduced TNF-α secretion, but was not sensitive to IL-6 and PGE2 secretion (Figure 4E).

## Discussion

Exposure to long-term LD cycle phase shifts can lead to immune system disruptions and increased levels of chemicals associated with inflammation and oxidative stress (Wright et al., 2015; Amara et al., 2019; Schilperoort et al., 2020). Disruption of circadian rhythms can also lead to an increase in circulating monocytes and macrophages, which can lead to increased systemic or peripheral tissue inflammation (McAlpine et al., 2016). In this study, we found that chronic CRD can cause OA-like pathological changes in knee cartilage, which is similar to other studies. In a study of temporomandibular joint osteoarthritis (TMJ-OA), Chen et al. (2020) found that interference with circadian rhythms with an improved multiplatform approach in Wistar rats resulted in OA-like lesions in mandibular cartilage. Meng et al. found similar OA-like pathological changes in knee cartilage of wild-type C57BL/6J mice, with cartilage surface fibrosis (Kc et al., 2015). However, the mechanism of the inflammation increase has not been clarified. Some studies proposed that CRD may exacerbate mice colitis by promoting mucosal immune dysregulation or intestinal necroptosis through dysactivation of the NF-κB pathway (Preuss et al., 2008; Pagel et al., 2017). Therefore, further studies *in vivo* or KEGG enrichment analysis are needed.

Core clock genes dominate the rhythm balance of tissues, among which BMAL1 can regulate the existence of several other clock genes, and plays a vital role in maintaining the normal function and integrity of body (Koronowski and Sassone-Corsi, 2021). Cartilage has its own peripheral biological clock, and is regulated by the central circadian clock. BMAL1 is stably expressed in healthy cartilage, but abnormally decreased in OA cartilage (Dudek et al., 2016; Akagi et al., 2017), which has become one of the characteristics of OA. On the contrary, abnormal expression of rhythm genes can also be one of the inducements to the occurrence of OA. This study found that both ACLT and CRD reduced the expression of BMAL1 in chondrocytes, which is also consistent with the research conclusion of Chen et al. (2020). They found that disruption of circadian rhythm resulted in decreased expression of BMAL1 in mandibular cartilage, which may be one of the causes of MMPs/TIMPs regulation imbalance. Systemic or cartilage-specific elimination of BMAL1 could impair the circadian rhythm of postpartum mice, showing significant loss of prosodic expression of several genes associated with cartilage, and distinctly elevated expression of MMP-3, MMP-13, VEGF, and Runx2 (Zhao et al., 2018; Ma et al., 2019). This suggests that inflammation and external stimuli disrupt the cartilage clock, possibly accelerating matrix degradation by activating some inflammatory pathways. In this experiment, the expression of MMP-3, MMP-13 and ADAMTS-4 in OA and CRD cartilage was significantly increased along with the decrease of BMAL1 protein expression, which proved the anabolism disorder of cartilage. Kc et al. already proposed that the OA-like pathological feature in mouse cartilage caused by CRD may be related to the activation of PKCδ-Erk-Runx2/NFκB and β-catenin signaling pathways (Kc et al., 2015). Based on this, we conducted *in vitro* studies targeting the β-catenin signaling pathway.

Wnt/β-catenin signaling pathway, one of the most studied pathways in OA, is closely related to chondrocyte differentiation, proliferation and apoptosis, and plays a crucial role in cartilage catabolism and regeneration (Onuora, 2017). Zhou et al. (2013) in their study of the therapeutic effect of tetrandrine on OA found that inhibiting the Wnt/β-catenin signaling pathway effectively reduced the expression of MMPs, and increased the expression of TIMP-1. Therefore, inhibiting the activation of Wnt/β-catenin may be an effective treatment for OA (Li et al., 2014). We demonstrated the dramatical increase of β-catenin expression in OA rat cartilage, and the pathway was significantly activated in cultured chondrocytes stimulated with IL-1β, highlighting the role of this pathway in OA. After XAV-939 inhibited this pathway, MMPs and inflammatory factors in chondrocytes were significantly reduced, indicating that inflammation of chondrocytes was dampened. One study found that β-catenin-induced degradation of PER2 increased CRD in intestinal mucosa of *Apc* ^*Min/+*^ mice (Yang et al., 2009); in exudative age-related macular degeneration (AMD) and autism spectrum disorder (ASD), the increase of the canonical WNT/β-catenin pathway is enhanced by the dysregulation of circadian rhythms (Vallée et al., 2020; Vallée et al., 2020). These evidences show that Wnt/β-catenin signaling pathway is regulated by circadian rhythm/circadian rhythm genes in many diseases. Specially, some studies have shown that BMAL1 can regulate Wnt/β-catenin pathway (Karantanos et al., 2014; Mao et al., 2020). Inhibition of BMAL1 expression leads to abnormal fat regulation through Wnt/β-catenin signaling pathway, resulting in increased adipogenesis in mice (Guo et al., 2012). Various Wnt-related proteins, such as Wnt10α, decreased in *BMAL1* ^*−/−*^ mice (Janich et al., 2011). Combined with the previous description, BMAL1 interacts synergistically with the Wnt/β-catenin signaling pathway in most diseases. Interestingly, an opposite result was obtained in our experiment. We found that OA and CRD significantly reduced the expression of BMAL1 in cartilage, but dramatically activated the Wnt/β-catenin signaling pathway. The *in vitro* studies also confirmed the results.

**FIGURE 5:**
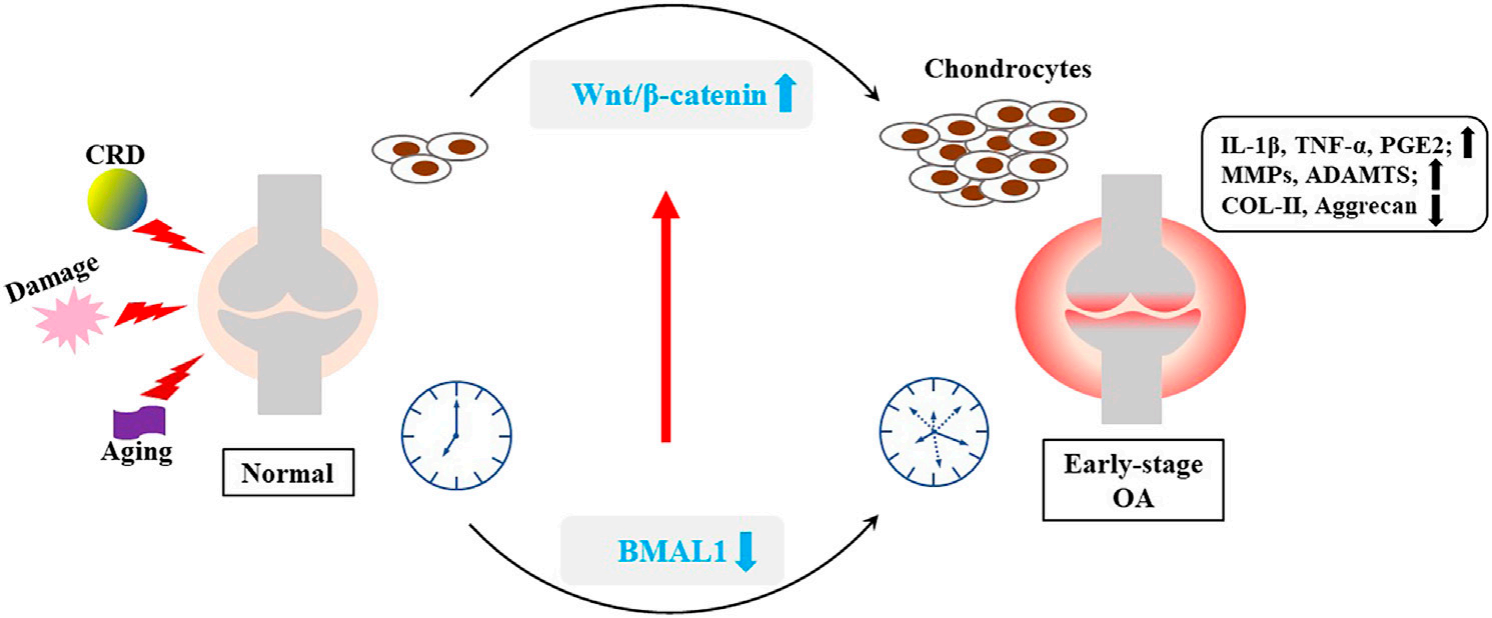
Interaction between BMAL1 and Wnt/β-catenin. Some internal and external factors, such as mechanical injury, aging and CRD, can stimulate inflammation of the knee cartilage, and disrupt the cartilage clock and matrix metabolism.

These present a question that whether the interaction mechanisms of BMAL1 and Wnt pathways are different in different microenvironments? And the Wnt/β-catenin pathway plays a different role? There are several possible explanations for the current contradictory findings. Firstly, The environment of cell proliferation is different. Some injuries, such as mechanical injury OA, compensatory cell proliferation is intense; The Wnt/β-catenin pathway is also significantly activated during vigorous periods of normal or abnormal proliferation of tumor or stem cells. Clock genes may play different roles in different contexts. In addition, the mechanism of Wnt/β-catenin pathway in OA is still unclear. Liu et al. presented a novel therapy in alleviating OA by activating the Wnt/β-catenin signaling pathway (Liu et al., 2019). Therefore, we temporarily speculated that the purpose of cell proliferation is discrepancy in the early and late stages of OA development, which may not be significantly related to the abnormal decrease of clock genes in OA. Furthermore, according to the phenomenon that low expression of BMAL1 in chondrocytes can also activate the Wnt/β-catenin pathway, it may be related to tissue or cell specificity. We have previously hypothesized that the clock is cell-specific (Song et al., 2021), therefore it is possible that clock genes may be abnormally expressed differently in different environments stimulated by inflammation or other cytokines for different cells (chondrocytes or tumor cells). In conclusion, the interaction between BMAL1 and Wnt/β-catenin signaling pathway needs further studies *in vitro* (Figure 5).

Additionally, we found that long-term CRD significantly accelerated weight gain in rats from Week 10, which was consistent with the results of Kc et al. (2015). Studies have shown a parallel relationship between increased nighttime lighting and obesity and metabolic disorders (Fonken and Nelson, 2014). CRD leads to abnormal hormone secretion, and then causes imbalance of the generation and decomposition of fat (Fonken and Nelson, 2014), indicating that CRD is one of the important causes of obesity. Different patterns of chronic jet lag (CJL) had diverse effects on rodent growth. A mount of systematic reviews has evaluated that shift work carried an extremely high risk of obesity (Sun et al., 2018; Zhang et al., 2020). The reason was that disruption of circadian rhythm would lead to a series of metabolic disorders such as hyperglycemia, hyperlipidemia, and hypoinsulinemia (Bass and Takahashi, 2010; Maury, 2019; McHill and Wright et al., 2017).

## Data Availability

The original contributions presented in the study are included in the article/Supplementary Material, further inquiries can be directed to the corresponding author.

## Supplementary Material

Supplementary Figure S1 Chondrocyte morphology and siBMAL1 transfection identification. **(A)** Toluidine blue staining of the secondary generation chondrocytes. Mag, 100×. **(B)** Immunofluorescent staining of COL2A1 in secondary generation chondrocytes. COL2A1, red; DAPI, blue. Mag, 400×. **(C)** Results of transfection of siBMAL1 into chondrocytes. Mag, 200×.

Supplementary Figure S2 Efficiency of silencing BMAL1 by siRNA. **(A)** Protein expression levels of BMAL1 in chondrocytes after transfecting three siBMAL1s. **(B)** Protein expression levels of BMAL1 in chondrocytes after transfecting the hybrid siBMAL1. **(C)** mRNA levels of BMAL1 in chondrocytes after transfecting the hybrid siBMAL1. All experiments are performed in triplicate, and the results are expressed as the mean±SEM. ***p*<0.01 vs. control group.
