# Supplementary figures and images for: Chronic Circadian Rhythm Disturbance Accelerates Knee Cartilage Degeneration in Rats Accompanied by the Activation of the Canonical Wnt/β-Catenin Signaling Pathway
Source: Front Pharmacol. 2021 Nov 11;12:760988. doi: 10.3389/fphar.2021.760988 (PMC8632052; doi:10.3389/fphar.2021.760988)

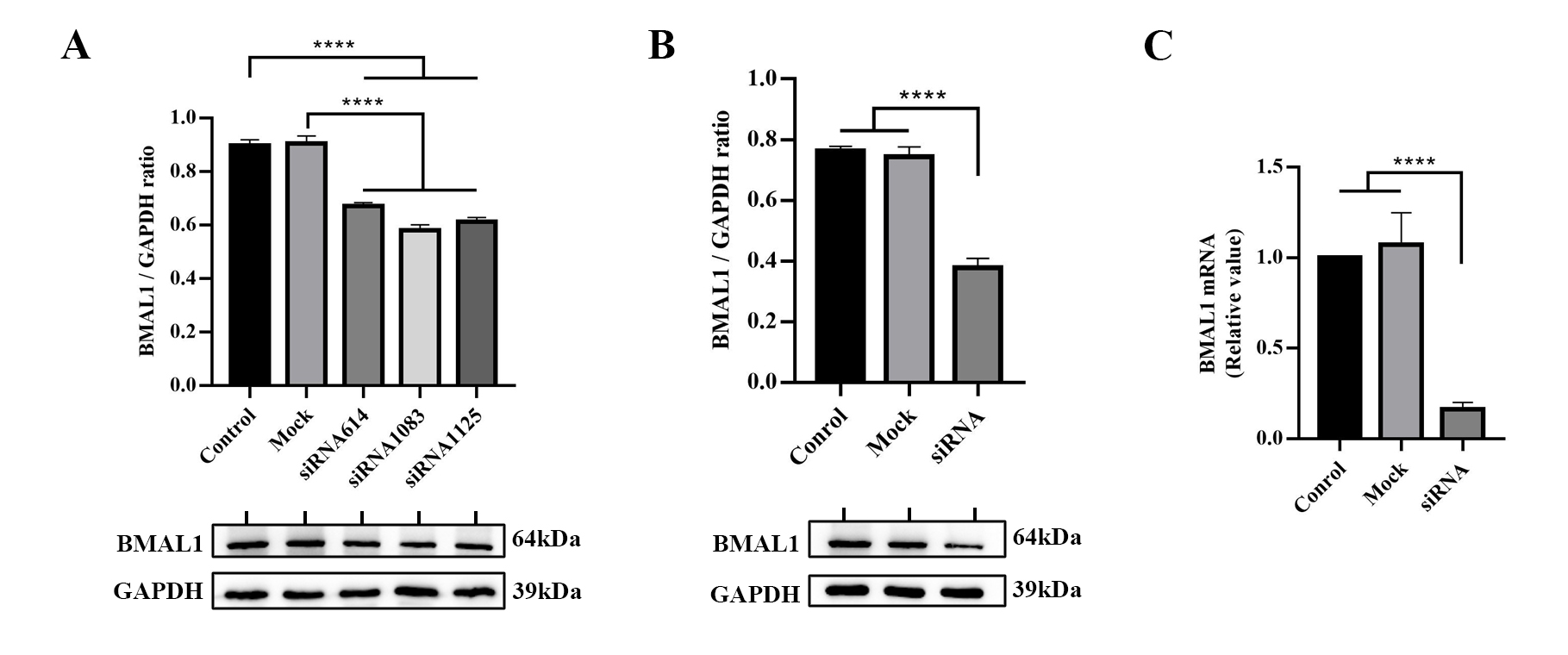

Supplement: Supplementary file 1 [file Image2.JPEG]

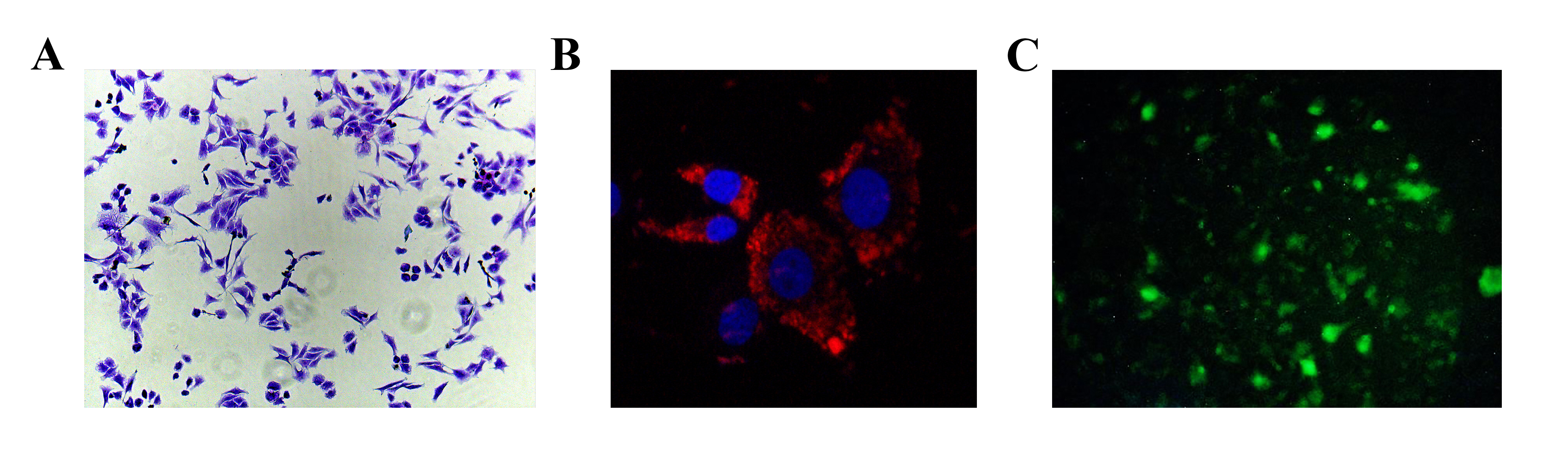

Supplement: Supplementary file 2 [file Image1.tif]
